# Changes in the Hematological Variables in Pigs Supplemented With Yeast Cell Wall in Response to a *Salmonella* Challenge in Weaned Pigs

**DOI:** 10.3389/fvets.2019.00246

**Published:** 2019-07-24

**Authors:** Nicole C. Burdick Sanchez, Jeffery A. Carroll, Jimmie R. Corley, Paul R. Broadway, Todd R. Callaway

**Affiliations:** ^1^USDA, ARS, Livestock Issues Research Unit, Lubbock, TX, United States; ^2^Phileo-Lesaffre Animal Care, Milwaukee, WI, United States; ^3^USDA, ARS, Food and Feed Safety Research Unit, College Station, TX, United States

**Keywords:** hematology, metabolism, pig, *Salmonella*, yeast cell wall

## Abstract

Stressors experienced by pigs at weaning may negatively impact health and productivity. Thus, supplements that enhance pig immunity during the early post-weaned period are of great interest to the swine industry. The objective of this experiment was to evaluate the performance and hematological responses of weaned pigs supplemented with yeast cell wall (YCW) when challenged orally with *Salmonella Typhimurium*. Weaned pigs were assigned to one of three treatments for 22d (*n* = 13/treatment): Control diet, which was a non-medicated starter diet (Control); Control diet supplemented with YCW at 250 mg/kg BW (YCW250; Phileo Lesaffre Animal Care, Milwaukee, Wisconsin, USA); and Control diet supplemented with YCW at 500 mg/kg BW (YCW500). On d19 blood samples were collected from −6 to 72 h relative to oral *Salmonella Typhimurium* (1 × 10^6^ cfu/pig) challenge. Gain:feed was greater (*P* = 0.01) in YCW250 treatment compared to both Control and YCW500 pigs. Baseline intraperitoneal temperature was greater (*P* < 0.001) in YCW250 pigs than Control or YCW500 pigs. There was a treatment x time interaction for the change in intraperitoneal temperature (*P* < 0.01), post-challenge cortisol, white blood cell counts (WBC), neutrophils, and neutrophil:lymphocyte ratio (*P* ≤ 0.03). Control pigs had greater (*P* < 0.05) cortisol concentrations than both YCW-supplemented groups at 0 h, but Control pigs had reduced (*P* < 0.05) cortisol compared to YCW500 pigs at 24 and 30 h post-challenge. Control pigs had greater (*P* < 0.05) WBC counts than both YCW-supplemented groups 6 and 12 h post-challenge, and YCW250 pigs had reduced (*P* < 0.01) WBC counts than Control and YCW500 pigs 18 h post-challenge. Neutrophil counts were greater (*P* < 0.05) in Control pigs than both YCW-supplemented groups at 6 and 12 h post-challenge and were greater (*P* = 0.02) than YCW250 pigs at 18 h post-challenge. Lymphocytes were greater (*P* < 0.001) in Control and YCW500 pigs pre- and post-challenge compared to YCW250 pigs. Control pigs had the greatest (*P* < 0.001) monocyte counts compared to YCW treatments. There was no effect of yeast supplementation on fecal shedding or *Salmonella* counts in the rectum, colon or cecum (*P* ≥ 0.05). While some differences were observed in intraperitoneal temperature and some hematological variables, data suggests there were minimal effects of yeast supplementation on the acute immune response to *Salmonella* challenge.

## Introduction

One of the most difficult time periods in swine production with regards to animal health is weaning, due to weaning stress, as well as the declining supply of maternal antibodies which can result in immunosuppression (1). Furthermore, swine production over the past three decades has focused on increasing lean muscle deposition, which has resulted in a decline in energy available for optimal function of the immune system, leaving pigs more susceptible to disease (2, 3). The subsequent increase in disease incidence following weaning further decreases growth, reducing the growth potential of pigs throughout the post-weaning period (4). Therefore, the nursery stage in swine is a critical area in which improvements need to be made to enhance the health and well-being of pigs.

Further complicating the management of swine health is the increased consumer pressure to produce a more natural product. Increased regulations on the use of antimicrobials, such as the Veterinary Feed Directive, add to the pressure, and have stimulated producers to find alternative management strategies that improve animal health while maintaining productivity. Feed additives offer many advantages, are easily incorporated into swine management procedures, and there are a large variety of products available to producers that claim to improve animal health. Live yeast and yeast cell wall products have the potential to enhance the acute phase response to various immune challenges (5–7). Recent studies in cattle have found variations in the immune and metabolic responses between live yeast and yeast cell wall, and also between different strains and products (8–11). Additionally, Collier et al. (12) found that supplementation of young pigs with *Saccharomyces cerevisiae* spp. *boulardii* enhanced average daily gain (ADG), reduced endotoxin-induced mortality and increased total white blood cell counts. The difference in circulating white blood cells is interesting and suggests a change in the inflammatory status of the pigs, potentially allowing the pigs to be more responsive to pathogenic infections. However, there is limited information on the effect of yeast products on leukocyte populations, particularly in response to pathogenic challenges.

There are several ways in which yeast are believed to improve immunity. Both live yeast and yeast cell wall have been demonstrated to directly bind pathogenic bacteria, thereby preventing the binding of the bacteria to the intestine and preventing pathogenic actions, thus potentially influencing host-microbiome interactions (13). Additionally, components of the cell wall, such as mannan oligosaccharides, may bind directly to the intestinal wall, and thus prevent the binding of bacteria to host epithelial cells (5, 14, 15), and may directly affect the growth of bacteria within the gut, altering the pathogenicity of the microbiome (16). Both of these actions would suggest a reduction in inflammation in the gut which may alter other immune parameters. Further, components of the cell wall of yeast, such as β-glucans and mannan oligosaccharides, have been demonstrated to alter immune function (5, 17), through activation of pattern recognition receptors and other responses, although the immune response elicited may be influenced by the overall health status of the animal (18). Approximately 70% of immune tissues are associated with the gastrointestinal tract (19). Reduction in inflammation within the gut may result in changes in other immune parameters, allowing for improved immune responses. Further, reduction in gut inflammation may improve gut integrity, reduce pathogen migration from the gastrointestinal tract, thus reducing overall systemic inflammation (20). However, much of this is speculation based on human and rodent models and has not been investigated in livestock species. Based on the potential effects of yeast products on enhancing immunity, it was hypothesized that supplementation with yeast cell wall would improve growth while altering populations of various circulating leukocyte populations and reducing bacteria concentrations within the gastrointestinal tract. Therefore, the objective of this study was to evaluate the performance and changes in leukocyte populations and tissue and digesta *Salmonella* concentrations of newly-weaned pigs supplemented with yeast cell wall products when orally challenged with *Salmonella enterica* serotype *Typhimurium*. *Salmonella* is one of the major pathogens associated with post-weaning diarrhea in pigs, with Typhimurium being the serotype most prevalent in swine (21). The use of a live *Salmonella* challenge in weaned pigs was used in this study to mimic post-weaning Salmonellosis in a controlled manner that produced mild to moderate morbidity in order to determine any potential effects of yeast cell wall supplementation.

## Materials and Methods

### Experimental Design

All experimental procedures were in compliance with the *Guide for the Care and Use of Agricultural Animals in Research and Teaching* and approved by the Institutional Animal Care and Use Committee at the Livestock Issues Research Unit.

**Figure 1:**
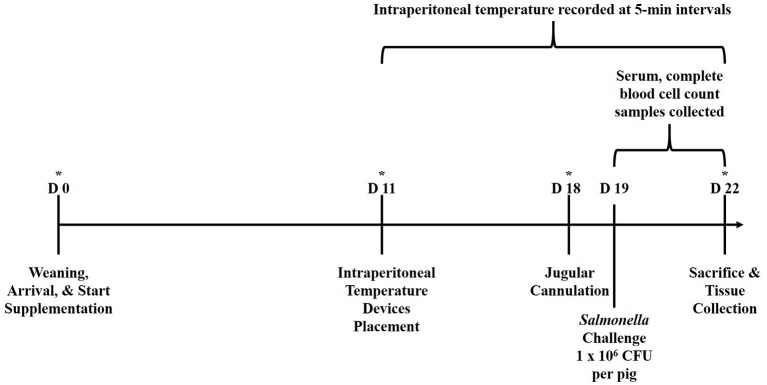
Graphical representation of the study experimental design relative to d of study. Pigs were ~21 d of age at the time of weaning (d 0). *Represents time points in which performance data (pig and feeder weights) were collected. Serum and CBC samples were collected at 6-h intervals from −6 to 72 h relative to *Salmonella* challenge at 0 h.

The experimental design is outlined in Figure 1. Newly weaned crossbred pigs (*n* = 39; barrows, ~21 d of age) were acquired from a commercial swine producer in Kansas, USA and transported approximately 480 km to the Livestock Issues Research Unit's Liberty Farm facility in Lubbock, Texas, USA. Pigs were housed in an enclosed, environmentally-controlled facility in individual stainless-steel pens (1.2 × 0.6 m) equipped with stainless steel feeders and nipple waterers. Pigs were weighed upon arrival (d 0) and balanced by body weight (BW) to one of three treatment diets (*n* = 13/treatment): (1) Control, fed a non-medicated starter ration; (2) fed the Control ration supplemented with yeast cell wall at 250 mg/kg BW (YCW250; Phileo Lesaffre Animal Care, Milwaukee, Wisconsin, USA); or (3) fed the Control ration supplemented with yeast cell wall at 500 mg/kg BW (YCW500). The YCW product used in the study was a commercially available product that was a proprietary combination of two cell walls from *Saccharomyces cerevisiae* (C-Wall, Phileo Lessaffre Animal Care). Pigs were provided corn/soybean meal-based treatment rations that was formulated to meet or exceeded NRC recommendations and water *ad libitum* for 22 d. Pigs and feeders were weighed weekly, and feed was added to feeders as needed to allow for *ad libitum* feed intake. On d 11, pigs were anesthetized and a small incision (2 to 2.5 cm) was made in the lower abdominal region for the placement of an indwelling temperature recording device (Star-Oddi DST micro-T; MeterMall USA, Marysville, Ohio, USA) into the peritoneal cavity which measured intraperitoneal temperature continuously every 5-min until the completion of the study (7). Specifically, pigs were placed ventral side up on a v-trough and mask-induced with 5% isoflurane and an oxygen flow rate of 2.0–3.0 L/min. Once pigs were completely anesthetized, isoflurane was reduced to 2.0–3.0% to maintain the pigs in an anesthetized state. At this point, 0.5 mL of lidocaine was applied subcutaneously. Next, the lower 1/3 of the abdomen, posterior to the navel, was surgically prepped with three gross scrubs of a diluted betadine solution, followed by a 70% ethanol wipe. A 2 to 2.5 cm incision was then made through the skin and muscle layers using a scalpel, lateral to the midline between the second and third teats relative to the posterior of the pig. The temperature recording device was then aseptically placed into the intraperitoneal cavity, and the muscle was sutured close, followed by the skin layer. The entire procedure was completed in <10 min. Intraperitoneal temperature was measured from the point the last pig had the temperature device placed until collection of the last sample, which encompassed 176 h prior to the challenge and 72 h following the challenge.

On d 18, pigs were anesthetized and fitted with indwelling jugular catheters using a non-surgical method (22). The following day (d 19), pigs were orally inoculated with an overnight Tryptic Soy Broth (TSB)-grown culture that was resuspended in fresh TSB (Difco, Detroit, Michigan, USA) to reach a challenge dose of 1 × 10^6^ CFU/pig at 0 h. The challenge dose was expected, as observed in previous studies at the Food and Feed Safety Research Unit (FFSRU; unpublished data), to produce a mild salmonellosis that resulted in increased diarrhea, lethargy, and reduced feed intake (moderate morbidity), while not resulting in mortality. *Salmonella enterica Typhimurium* originally isolated from swine and maintained in the FFSRU culture collection was repeatedly grown by 10% (vol/vol) transfer in anoxic (85% N_2_, 10% CO_2_, 5% H_2_ atmosphere) Tryptic soy broth (TSB) medium at 37°C. This strain was selected for resistance to novobiocin and nalidixic acid (20 and 25 μg/mL, respectively) by repeated transfer and selection in the presence of sub-lethal concentrations of each antibiotic. This resistant phenotype was stable through multiple unselected transfers in batch culture and through repeated culture vessel turnovers in continuous culture (data not shown). Overnight cultures were harvested by centrifugation (7,500 x g, 10 min) and cell pellets were re-suspended in TSB medium. One 4.5 mL whole blood sample was collected in Starstedt tubes containing no additive (Starstedt Inc., Newton, North Carolina, USA) at 6-h intervals from −6 to 72 h relative to the oral *Salmonella* challenge. Samples were allowed to clot at room temperature for 30 min prior to centrifugation at 1,500 g for 20 min at 4°C. Isolated serum was stored at −80°C until analyzed for cortisol, glucose and non-esterified fatty acid (NEFA) concentrations. A second 4-mL sample was collected in a vacutainer tube (Fisher Scientific, Pittsburgh, Pennsylvania, USA) containing EDTA for determination of red blood cell, hemoglobin, hematocrit, platelet, total white blood cell (WBC) and differential counts using a Procyte Dx Hematology Analyzer (IDEXX, Westbrook, Maine, USA) using porcine-specific analogs. Fecal samples were collected at 0, 24, and 48 h following challenge and quantified for *Salmonella* concentrations (described below). Following collection of the 72 h blood samples, all pigs were humanely euthanized, and tissue samples from the ileocecal lymph node, and tissue and digesta samples from the cecum, colon, and rectum were collected for quantification of *Salmonella* concentrations. The rectum sample collected at 72 h post-challenge was assumed as the 72-h fecal sample.

### Serum Analysis

All serum samples were analyzed in duplicate and serum cortisol concentrations were determined using a commercially-available, porcine-specific enzyme immunoassay kit (Abnova, Taiwan) according to the manufacturers' directions. The intra- and inter-assay coefficients of variation were 13.2 and 16.5%, respectively. Serum glucose concentrations were determined by modification of the enzymatic Autokit Glucose (Wako Diagnostics, Richmond, Virginia, USA) to fit a 96-well format as previously described (23). Briefly, 300 μL of prepared working solution was added to 2 μL of serum or prepared standards in a 96-well plate. Plates were incubated at 37°C for 5 min and absorption was recorded at 505 nm. The plate reader used for this assay has an incubating and timing feature and therefore ensured that the sample absorbances were read immediately following the 5-min incubation. Concentrations of glucose were determined by comparing unknown samples to a standard curve of known glucose concentrations. The intra- and inter-assay coefficients of variation were 8.5 and 9.6%, respectively. Serum NEFA concentrations were determined by modification of the enzymatic HR Series NEFA-HR (2) assay (Wako Diagnostics, Richmond, Virginia, USA) to fit a 96-well format as previously described (23). Briefly, 200 μL of the prepared Color Reagent A was added to 5 μL of serum or prepared standards in a 96-well plate. Plates were incubated at 37°C for 5 min and then the absorbance was read using a plate reader at 550 nm. Next, 100 μL of prepared Color Reagent B was added to all wells on the 96-well plate. Plates were incubated for an additional 5 min and read for a second time using a plate reader at 550 nm. The plate reader used for this assay has an incubating timing feature which therefore ensured that the sample absorbances were read immediately following the 5-min incubation. A final absorbance was obtained by subtracting the first reading, which was multiplied by a factor of 0.67 to account for volume changes, from the second reading. Final absorbance values were used for all calculations (i.e., standard curve, sample concentrations). Concentrations of NEFAs were determined by comparing unknown samples to a standard curve of known NEFA concentrations. The intra- and inter-assay coefficients of variation were 12.9 and 15.5%, respectively.

### Fecal and Tissue *Salmonella* Analysis

Prior to experimental infection, fecal samples from all pigs were collected and enriched to confirm the absence of *Salmonella* and to ensure that no bacteria with similar morphology resistant to nalidixic acid were present. For qualitative enrichment of *Salmonella*, 3 g of aseptically collected tissue or digesta were added to tubes containing 27 mL of Tetrathionate broth (Difco Laboratories, Detroit, Michigan, USA) and incubated at 37°C for 24 h. After this incubation, 200 μL of the Tetrathionate enrichment were added to 5 mL Rappaport-Vassiliadis R10 broth (24) and incubated an additional 24 h at 42°C before being streak-plated onto brilliant green agar (BGA) supplemented with novobiocin (25 μg/mL) and nalidixic acid (20 μg/mL). Plates were incubated for 24 h at 37°C; colonies that exhibited typical *Salmonella* morphology (pink/white, opaque, circular, entire, convex colonies of a medium size surrounded by a brilliant red medium [alkaline]) were individually picked for further biochemical characterization. Putative *Salmonella* colonies were picked and inoculated onto Triple Sugar Iron (TSI) agar slants and Lysine Iron agar (LIA) slants (Difco Laboratories). Each slant was incubated at 37°C for 24 h. *Salmonella*-positive samples were confirmed by slide agglutination using SM-O antiserum poly A-I and V-I, and group C1 factors. *Salmonella* isolates were stored in glycerol and TSB at −80°C until confirmatory serotyping was performed by the National Veterinary Services Laboratory (NVSL) in Ames, IA.

### Statistical Analysis

Prior to data analysis, intraperitoneal temperature data were averaged into 1-h intervals. With the exception of performance and *Salmonella* count data, all data were analyzed separately in two time blocks, Pre-challenge (−6 and 0 h), and Post-challenge (0 to 72 h). Performance, serum, and hematology data were analyzed using the MIXED procedure of SAS, specific for repeated measures. Treatment, time, and treatment x time were included as fixed effects, and pig within treatment was included as the subject. When significant, specific treatment differences were separated using the PDIFF option in SAS. *Salmonella* tissue and fecal count data were analyzed in SAS using Proc Glimmix of SAS at an α = 0.05 utilizing the Tukey option for mean separation. *P* < 0.05 was considered significant, and 0.05 ≤ *P* ≤ 0.10 was considered a tendency.

## Results

### Performance and Intraperitoneal Temperature

Pig body weight was not different between treatments (*P* = 0.49) but increased over time, as expected (*P* < 0.001). There was a tendency (*P* = 0.07) for ADG to be reduced in YCW500 compared to YCW250, while Control ADG was intermediate. Additionally, there was a tendency (*P* = 0.08) for a treatment effect on feed intake, with feed intake reduced in YCW250 pigs compared to Control, and with YCW500 pigs being intermediate. There was a treatment effect (*P* = 0.01) on gain:feed which was greater in YCW250 compared to both Control and YCW500 pigs. Performance data are summarized in Table 1.

**Table 1:** Performance parameters measured in pigs fed a control (Control) diet or supplemented with a yeast cell wall at 250 mg/kg (YCW250) or 500 mg/kg (YCW500), with administration of an oral *Salmonella Typhimurium* challenge on d 19.

		**Day of study**					***P*****-value**		
**Variable**	**TRT**	**0**	**11**	**18**	**22**	**SEM**	**TRT^**c**^**	**Time**	**Interaction^**d**^**
Weight (kg)	Control	7.13	7.70	10.75	11.96	0.30	0.49	**<0.001**	0.68
	YCW250	7.04	7.65	10.14	11.79	0.32			
	YCW500	7.35	7.85	10.10	11.34	0.30			
ADG (kg/d)^a^	Control		0.05	0.43	0.30	0.03	0.07	**<0.001**	0.11
	YCW250		0.06	0.36	0.40	0.03			
	YCW500		0.04	0.31	0.29	0.03			
Feed Disappearance (kg)	Control		1.69	3.70	2.48	0.19	0.08	**<0.001**	0.80
	YCW250		1.57	3.10	2.13	0.21			
	YCW500		1.57	3.38	2.24	0.20			
G:F (kg)^b^	Control		0.28	0.83	0.49	0.07	**0.01**	**<0.001**	0.35
	YCW250		0.40	0.79	0.76	0.08			
	YCW500		0.21	0.66	0.53	0.07			

a *ADG, average daily gain*.

b *G:F, gain to feed*.

c *TRT, Treatment*.

d *Interaction, Treatment x time*.

*Bold text represents significant values*.

**Figure 2:**
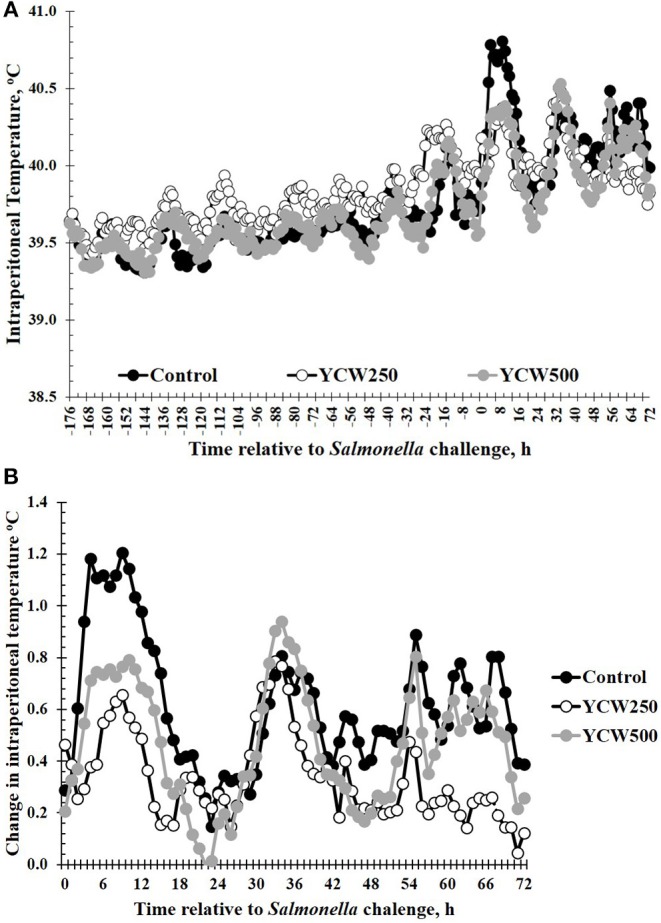
**(A)** The intraperitoneal temperature response and **(B)** change in intraperitoneal temperature in pigs fed a control (Control) diet or supplemented with a yeast cell wall at 250 mg/kg (YCW250) or 500 mg/kg (YCW500), following administration of an oral *Salmonella Typhimurium* challenge on d 19. **(A)** SEM ± 0.12°C for all treatment groups; **(B)** SEM ± 0.01°C for all treatment groups.

Intraperitoneal temperature over the 176 h (7.3 d) prior to the *Salmonella* challenge was greater (*P* < 0.001) in the YCW250 pigs compared to Control and YCW500 pigs (Figure 2A; Table 2). There was a treatment x time interaction for post-challenge intraperitoneal temperature (Figure 2A; Table 4). Specifically, YCW250 pigs had reduced (*P* ≤ 0.05) intraperitoneal temperature than Control pigs from 3 to 15 h, 55 to 56 h, 61 to 63 h, and 67 to 69 h, yet YCW250 had greater intraperitoneal temperature than Control pigs at 30 h (*P* = 0.05). Additionally, YCW500 pigs had reduced (*P* ≤ 0.04) intraperitoneal temperature than Control pigs from 3 to 6 h, 8 to 10 h, and at 45 h post-challenge. Lastly, YCW250 pigs had greater (*P* ≤ 0.05) intraperitoneal temperature than YCW500 pigs from 20 to 22 h. Based on baseline treatment differences in intraperitoneal temperature, the post-challenge temperature data was analyzed as the change in intraperitoneal temperature relative to average baseline values. There was a treatment x time interaction for the change in intraperitoneal temperature (*P* = 0.002; Figure 2B; Table 4). Control pigs had a greater change in intraperitoneal temperature than YCW250 pigs from 3 to 17 h, at 38 h, from 55 to 58 h, from 61 to 64 h, and from 67 to 71 h. Control pigs also had greater change in intraperitoneal temperature than YCW500 pigs from 3 to 10 h, and at 45 h. Finally, the change in intraperitoneal temperature was greater in YCW500 than YCW250 pigs from 4 to 5 h, from 14 to 15 h, at 55 h, and from 61 to 69 h post-challenge.

**Table 2:** Intraperitoneal temperature and serum cortisol, glucose and non-esterified fatty acid (NEFA) concentrations measured in pigs fed a control (Control) diet or supplemented with a yeast cell wall at 250 mg/kg (YCW250) or 500 mg/kg (YCW500), prior to administration of an oral *Salmonella Typhimurium* challenge on d 19.

					**Pre-challenge** ***P*****-values**		
**Variable**	**Control^**1**^**	**YCW250**	**YCW500**	**SEM**	**TRT^**2**^**	**Time**	**Interaction^**3**^**
IP^4^ temperature (°C)	39.58^b^	39.75^a^	39.58^b^	0.01	**<0.001**	**<0.001**	1.00
Cortisol (ng/mL)	7.29	4.73	6.51	0.92	0.11	**0.004**	0.34
Glucose (mg/dL)	90.41	94.66	88.12	2.50	0.17	0.23	0.53
NEFA (mmol/L)	0.14	0.14	0.12	0.01	0.30	0.14	0.91

ab *Treatments with different superscripts within rows differ (P ≤ 0.05)*.

1 *Values for Control and YCW treatments represent average values across all time points*.

2 *TRT, Treatment*.

3 *Interaction, Treatment x time*.

4 *IP, intraperitoneal*.

*Bold text represents significant values*.

### Serum Analysis

No treatment differences were observed between groups for serum cortisol concentrations prior to challenge (*P* = 0.11; Table 2; Supplementary Table 1). However, following the *Salmonella* challenge, there was a treatment x time interaction (*P* = 0.03; Table 4; Supplementary Table 3) such that cortisol concentrations were greater in Control pigs than both YCW supplemented groups at 0 h, but was reduced in Control pigs compared to YCW500 pigs at 24 and 30 h. However, it should be noted that throughout the challenge serum cortisol concentrations remained very low (i.e., below 10 ng/mL) suggesting as previously observed that cortisol may not be a good indicator of infection.

Serum glucose concentrations did not differ between treatments prior to the challenge (*P* = 0.17; Table 2; Supplementary Table 1). Following *Salmonella* challenge there was a treatment x time interaction (*P* = 0.01) for serum glucose concentrations (Table 4; Supplementary Table 3). Specifically, glucose was greater in YCW250 than Control pigs at 6 h (*P* = 0.02), yet at 60 h Control pig glucose concentration was greater than in YCW250 pigs (*P* = 0.02). Additionally, YCW250 pigs had greater glucose concentrations at 18 h compared to YCW500 pigs, but by 30 h YCW500 pigs had greater glucose concentrations than did YCW250 pigs. Lastly, glucose concentrations were reduced in YCW500 pigs at 72 h (*P* ≤ 0.02) compared to the other treatment groups.

Similar to glucose, there was no treatment effect for serum NEFA concentrations prior to administration of *Salmonella* (*P* = 0.30; Table 2; Supplementary Table 1). After challenge, NEFA concentrations were decreased (*P* = 0.002) in both YCW treatment groups compared to Control pigs (Table 4; Supplementary Table 3). Additionally, there was a tendency (*P* = 0.07) for a treatment x time interaction such that NEFA concentrations were greater in Control compared to both YCW treatment groups at 6, 18, 24 (YCW500 only), and 72 h post-challenge.

### Hematology Analysis

**Figure 3:**
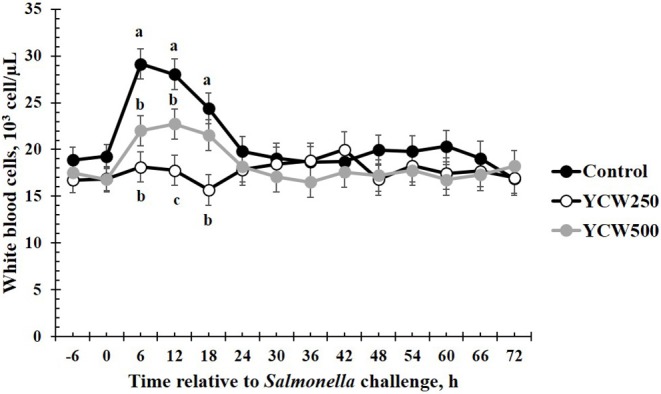
White blood cell concentrations measured in pigs fed a control (Control) diet or supplemented with a yeast cell wall at 250 mg/kg (YCW250) or 500 mg/kg (YCW500), following administration of an oral *Salmonella Typhimurium* challenge on d 19. ^abc^Treatments differ *P* < 0.02.

**Figure 4:**
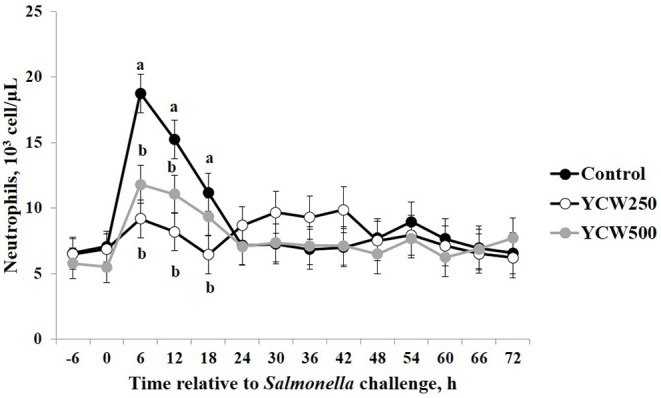
Neutrophil concentrations measured in pigs fed a control (Control) diet or supplemented with a yeast cell wall at 250 mg/kg (YCW250) or 500 mg/kg (YCW500), following administration of an oral *Salmonella Typhimurium* challenge on d 19. ^ab^Treatments differ *P* ≤ 0.04.

**Figure 5:**
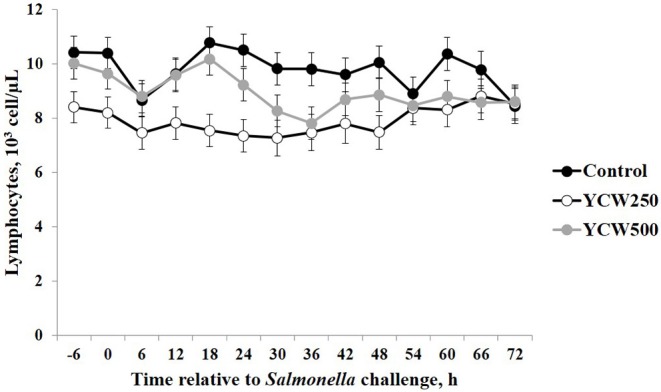
Lymphocyte concentrations measured in pigs fed a control (Control) diet or supplemented with a yeast cell wall at 250 mg/kg (YCW250) or 500 mg/kg (YCW500), following administration of an oral *Salmonella Typhimurium* challenge on d 19.

**Figure 6:**
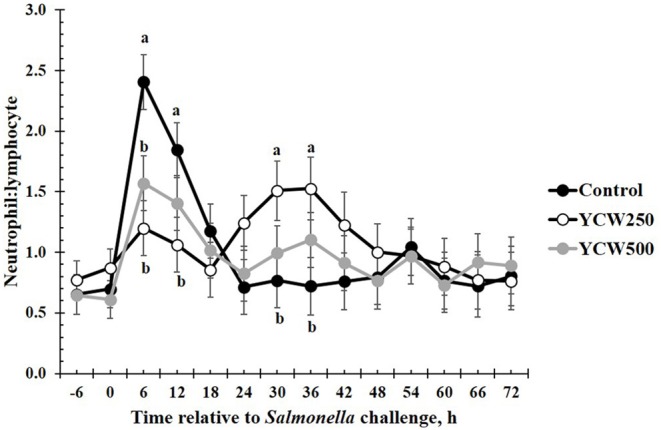
The neutrophil:lymphocyte ratio measured in pigs fed a control (Control) diet or supplemented with a yeast cell wall at 250 mg/kg (YCW250) or 500 mg/kg (YCW500), following administration of an oral *Salmonella Typhimurium* challenge on d 19. ^ab^Treatments differ *P* < 0.03.

Prior to administration of the *Salmonella* challenge, the only hematology variables that were affected by treatment were red blood cell (RBC), platelet, and lymphocyte concentrations (Table 3; *P* ≤ 0.05). For RBC, the YCW500 pigs had greater pre-challenge concentrations than Control pigs, while both YCW treatment groups had greater platelet concentrations than Control pigs. Lymphocytes were greater in Control and YCW500 treatments compared to YCW250 pigs (*P* = 0.002; Table 3). Pre-challenge time effects for hematology variables are summarized in Supplementary Table 2. Following challenge administration, there were treatment effects for RBC, hemoglobin, hematocrit and platelets (Table 5; *P* ≤ 0.002), and all four variables, except for platelets, decreased over time (*P* < 0.001; Supplementary Table 4). Red blood cell concentrations were greater in YCW250 pigs than Control pigs. Hemoglobin and hematocrit concentrations were greater in Control and YCW250 pigs compared to YCW500 pigs. Platelet concentrations were greater in both YCW treatment groups than Control pigs. There was a treatment x time interaction for total white blood cell concentrations (*P* = 0.03; Figure 3). Specifically, Control pigs had greater white blood cell concentrations at 6 h, while all three treatments differed from each other at 12 h, yet YCW250 had reduced white blood cell concentrations at 18 h compared to Control and YCW500 pigs. Similar to white blood cells, there was a treatment x time interaction for neutrophil concentrations (*P* = 0.02; Figure 4). Neutrophils were greater in Control than YCW supplemented pigs at 6, 12, and 18 h (YCW250 only). Lymphocyte concentrations following *Salmonella* challenge were greater in Control and YCW500 pigs compared to YCW250 pigs (treatment *P* < 0.001; Figure 5). There was a treatment x time interaction (*P* = 0.03) for the neutrophil:lymphocyte ratio such that the ratio was greater in Control pigs than in either YCW treatment group at 6 h and was elevated in Control compared to YCW250 pigs at 12 h post challenge (Figure 6). Additionally, the neutrophil:lymphocyte ratio was greater in YCW250 pigs than Control pigs from 30 and 36 h post-challenge. Post-challenge monocytes were greatest in Control, followed by YCW250 and YCW500 pigs (treatment *P* < 0.001; Table 5; Supplementary Table 4). Eosinophils were not affected by treatment (*P* = 0.55), but basophil concentrations were greater in YCW250 compared to Control and YCW500 pigs (*P* < 0.001).

**Table 3:** Complete blood cell count parameters measured in pigs fed a control (Control) diet or supplemented with a yeast cell wall at 250 mg/kg (YCW250) or 500 mg/kg (YCW500), prior to administration of an oral *Salmonella Typhimurium* challenge on d 19.

					**Pre-challenge** ***P*****-values**		
**Variable**	**Control^**1**^**	**YCW250**	**YCW500**	**SEM**	**TRT^**2**^**	**Time**	**Interaction^**3**^**
Red blood cells (10^6^/μL)	5.28^b^	5.50^ab^	5.59^a^	0.09	**0.05**	0.22	0.87
Hemoglobin (g/dL)	8.69	8.84	8.83	0.13	0.64	0.26	0.84
Hematocrit (%)	28.20	28.59	28.51	0.46	0.82	0.23	0.83
Platelets (10^3^/μL)	324.86^b^	393.21^a^	401.17^a^	18.90	**0.01**	0.56	0.83
White blood cells (10^3^/μL)	19.06	16.80	17.16	0.95	0.20	0.95	0.90
Neutrophils (10^3^/μL)	6.82	6.68	5.64	0.85	0.55	0.83	0.94
Lymphocytes (10^3^/μL)	10.41^a^	8.30^b^	9.83^a^	0.41	**0.002**	0.67	0.96
N:L^4^	0.67	0.82	0.67	0.11	0.43	0.78	0.91
Monocytes (10^3^/μL)	0.05	0.17	0.12	0.11	0.31	0.76	0.97
Eosinophils (10^3^/μL)	0.24	0.27	0.28	0.05	0.60	0.15	0.60
Basophils (10^3^/μL)	0.03	0.03	0.01	0.01	0.34	0.96	0.17

ab *Treatments with different superscripts within rows differ (P ≤ 0.05)*.

1 *Values for Control and YCW treatments represent average values across all time points*.

2 *TRT: Treatment*.

3 *Interaction: Treatment x time*.

4 *N:L: Neutrophil:lymphocyte ratio*.

*Bold text represents significant values*.

**Table 4:** Intraperitoneal temperature, serum cortisol, glucose, and non-esterified fatty acid (NEFA) concentrations measured in pigs fed a control (Control) diet or supplemented with a yeast cell wall at 250 mg/kg (YCW250) or 500 mg/kg (YCW500), following administration of an oral *Salmonella Typhimurium* challenge on d 19.

					**Post-challenge** ***P*****-values**		
**Variable**	**Control^**1**^**	**YCW250**	**YCW500**	**SEM**	**TRT^**2**^**	**Time**	**Interaction^**3**^**
IP^4^ temperature (°C)	40.21^a^	40.05^b^	40.06^b^	0.01	**<0.001**	**<0.001**	**0.03**
IP temperature Change (°C)	0.61^a^	0.34^c^	0.46^b^	0.01	**<0.001**	**<0.001**	**0.002**
Cortisol (ng/mL)	3.96	4.11	4.68	0.26	0.09	**<0.001**	**0.03**
Glucose (mg/dL)	93.78	93.20	93.32	1.22	0.93	**<0.001**	**0.01**
NEFA (mmol/L)	0.17^a^	0.14^b^	0.14^b^	0.01	**0.002**	0.06	0.07

ab *Treatments with different superscripts within rows differ (P ≤ 0.05)*.

1 *Values for Control and YCW treatments represent average values across all time points*.

2 *TRT: Treatment*.

3 *Interaction: Treatment x time*.

4 *IP: intraperitoneal*.

*Bold text represents significant values*.

**Table 5:** Complete blood cell count parameters measured in pigs fed a control (Control) diet or supplemented with a yeast cell wall at 250 mg/kg (YCW250) or 500 mg/kg (YCW500), following administration of an oral *Salmonella Typhimurium* challenge on d 19.

					**Post-challenge** ***P*****-values**		
**Variable**	**Control^**1**^**	**YCW250**	**YCW500**	**SEM**	**TRT^**2**^**	**Time**	**Interaction^**3**^**
Red blood cells (10^6^/μL)	5.13^b^	5.30^a^	5.21^ab^	0.03	**0.002**	**<0.001**	0.81
Hemoglobin (g/dL)	8.37^a^	8.41^a^	8.16^b^	0.05	**0.001**	**<0.001**	0.93
Hematocrit (%)	27.60^a^	27.26^a^	26.54^b^	0.20	**<0.001**	**<0.001**	0.87
Platelets (10^3^/μL)	359.51^b^	392.86^a^	395.11^a^	6.70	**<0.001**	0.09	0.99
White blood cells (10^3^/μL)	21.01^a^	17.75^b^	18.44^b^	0.47	**<0.001**	**<0.001**	**0.03**
Neutrophils (10^3^/μL)	9.11^a^	7.96^ab^	7.80^b^	0.42	**0.05**	**<0.001**	**0.02**
Lymphocytes (10^3^/μL)	9.75^a^	7.88^c^	8.88^b^	0.17	**<0.001**	0.25	0.52
N:L^4^	1.10	1.07	0.98	0.07	0.61	**<0.001**	**0.03**
Monocytes (10^3^/μL)	1.79^a^	1.58^b^	1.44^c^	0.04	**<0.001**	**<0.001**	0.16
Eosinophils (10^3^/μL)	0.34	0.26	0.29	0.05	0.55	0.08	0.87
Basophils (10^3^/μL)	0.02^b^	0.07^a^	0.03^b^	0.01	**<0.001**	0.33	0.31

abc *Treatments with different superscripts within rows differ (P ≤ 0.05)*.

1 *Values for Control and YCW treatments represent average values across all time points*.

2 *TRT, Treatment*.

3 *Interaction, Treatment x time*.

4 *N:L, Neutrophil:lymphocyte ratio*.

*Bold text represents significant values*.

### Fecal and Tissue *Salmonella* Analysis

*Salmonella* was only detected in the ileocecal lymph nodes in 14 of the 39 pigs (4 Control, 5 YCW250, and 5 YCW500). There was no effect of treatment (*P* = 0.17) on fecal *Salmonella* counts collected daily through 72 h post-challenge (Table 6). However, there was an effect of time (*P* = 0.02), such that *Salmonella* counts decreased from 24 h to 48 h post-challenge. Additionally, there was a tendency (*P* = 0.09) for a treatment x time interaction, where YCW250 pigs had greater fecal *Salmonella* counts than YCW500 pigs at 24 h, and YCW500 pigs had greater fecal *Salmonella* counts than Control pigs at 48 h. There was no effect of treatment on *Salmonella* counts in the rectum, colon, or cecum (*P* ≥ 0.14; Table 6).

**Table 6:** Fecal and tissue *Salmonella* counts (log CFU/g) measured in pigs fed a control (Control) diet or supplemented with a yeast cell wall at 250 mg/kg (YCW250) or 500 mg/kg (YCW500), following administration of an oral *Salmonella Typhimurium* challenge on d 19.

**Variable**	**Control**	**YCW250**	**YCW500**	**SEM**	**TRT^**a**^**	**Time**	**Interaction^**b**^**
Fecal 24 h	1.15	1.98	0.95	0.37	0.17	0.02	0.09
Fecal 48 h	0.00	0.33	1.29	0.37			
Fecal 72 h^c^	0.78	1.11	1.12	0.37			
Colon	0.00	0.73	0.76	0.31	0.14		
Cecum	0.40	1.13	0.56	0.32	0.25		

a *TRT: Treatment*.

b *Interaction: Treatment x time*.

c *Rectum sample means are presented by the Fecal 72h sample; Rectal TRT P = 0.84*.

## Discussion

There were no treatment differences for fecal *Salmonella* shedding, nor in the *Salmonella* content of the cecum, colon or rectum. A study using a different challenge strain of *Salmonella Typhimurium* in pigs observed increased fecal shedding of *Salmonella* when fed a yeast fermentation product (25). Similar to the current study, another group of investigators found pigs supplemented with yeast as a source of mannan oligosaccharides had no effect on fecal bacterial counts, including *Salmonella* and E*. coli* (26). Therefore, it is possible that yeast products have minimal effects on the fecal shedding of *Salmonella* in pigs. Additionally, less than half of the ileocecal lymph nodes were positive for *Salmonella* in the current study. This suggests that either the inoculating concentration or strain pathogenicity was insufficient to overwhelm intestinal immunity and mesenteric lymph nodes allowing system migration. This would explain the limited intraperitoneal temperature response, as well as the limited effects on the hematological parameters measured in this study. While no differences in *Salmonella* shedding or tissue content were observed, it is possible that there were effects of the YCW supplement on other aspects of gut microbial populations, as has been observed in other species (18). Future studies may benefit from analyzing effects of YCW supplements on the entire gut microbiome, and the influence of any microbiome population changes on the overall health of the animal.

One of the earliest indicators of illness is reduced growth performance because animals that are sick typically reduce or eliminate feed intake. This was evident in the current study in which feed intake was reduced in all treatments following *Salmonella* challenge. Interestingly, the YCW500 treatment had reduced ADG, suggesting that the level of YCW supplementation may have been too high and may consequently have impaired performance. Previous studies utilizing pigs supplemented with a yeast fermentation product also found limited differences in performance (7). Studies using yeast cell wall extract from *Saccharomyces cerevisiae* found improved ADG in weaned pigs when fed at 0.05 to 0.15% of the diet (27). However, another study found no effects on performance when a yeast cell wall extract was fed to young pigs (28).

Beta-glucans, a component of the cell wall of yeast, have been demonstrated to have a role in pig growth. Specifically, β-glucans promote a healthy gut by increasing mucosal barrier functions through modulating the intestinal microbiota and supporting the immune system in the gut (29). Additionally, supplementation of pigs with *Saccharomyces cerevisiae boulardii* has increased villus height and crypt depth within the small intestine, while decreasing goblet cells (30, 31). Bontempo, Di Giancamillo (31) found an increase in the mucin content of goblet cells within the ileum, while observing a decrease in mucous layer thickness in yeast supplemented pigs compared to control pigs. The authors suggested that the greater mucous thickness may be a result of greater pathogenic bacteria in the gut of control pigs compared to those pigs supplemented with live yeast, and may also reduce nutrient absorption, and thus reduce growth in control pigs.

An increase in body temperature in response to a bacterial challenge is common, as fever enhances bacterial killing efficacy of the immune system (32). The intraperitoneal temperature response in the current study is similar in temporal pattern and magnitude to that of previous *Salmonella* challenge studies (33, 34). However, a study by Balaji et al. (35) demonstrated an increase in body temperature sooner, within 12 h of challenge, with a greater dose of *Salmonella Typhimurium*, whereas a *Salmonella*-induced increase was not observed until approximately 42 h post-challenge in the current study, as discussed further below. It is possible that the greater challenge dose resulted in increases in rectal temperature more quickly in the aforementioned study than in the current study. Additionally, there could be differences in the manner in which body temperature was measured, as the previous study utilized measuring rectal temperature every 6 h, which the current study utilized an indwelling temperature probe in the intraperitoneal cavity that measured temperature continuously every 5 min. The difference in intraperitoneal temperature prior to the challenge is of interest as it occurred in only one treatment group, the YCW250 treatment. Similar to the differences observed in performance, it suggests a potential benefit from the 250 mg/kg dose but not the 500 mg/kg dose, as Control and YCW500 had similar body temperatures prior to the challenge.

The post-challenge temperature response was very interesting. First, there was a rapid increase in intraperitoneal temperature immediately following administration of the *Salmonella* challenge (6 to 18 h). This response is likely a response to the stress associated with the dosing, as the pigs had to be picked up for the oral *Salmonella* administration. Data in pigs has demonstrated that restraint stress can result in an increase in body temperature (36). While one could question whether a stress response occurred, due to the lack of a cortisol response, it is important to note that serum samples were only collected every 6 h, which is sufficient time for any acute increase in cortisol resulting from handling to subside (37). If indeed there was an acute temperature response to the *Salmonella* challenge, it would be difficult to separate these effects from the effects of the dosing procedure in the current study. Acute effects of *Salmonella* have not been well-documented in the literature, as most affects appear to be observed at least 12 h post-challenge in swine. In support of this, a study conducted by Bellido-Carreras, Arguello (38) observed fecal shedding of *Salmonella* within 1-day post infection which subsequently decreased, while tissue *Salmonella* content increased from day 1 to day 2 post infection. Further, differences were observed in the timing of leukocyte subset infiltration into tissues. This suggests a distinction between fecal shedding of *Salmonella* and the tissue *Salmonella* colonization within the gastrointestinal tract during early infection. Samples for *Salmonella* enumeration in the current study were not collected until 3 days post infection, and thus the acute period following *Salmonella* infection is an area that requires further study.

The second interesting portion of the post-challenge temperature response was the increase in intraperitoneal temperature that occurred later in the challenge, beginning approximately 42 h post challenge until 71 h, particularly in the Control and YCW500 treatment groups. This response, as reported by other studies (33, 34), is likely a response to the *Salmonella* infection. While increases in body temperature are observed in Control and YCW500 groups, there was minimal change in intraperitoneal temperature in the YCW250 group. *Salmonella* is an intracellular pathogen, eliciting its effects once taken up by intestinal epithelial cells. This action activates the innate immune system, one pathway being through the action of flagellin recognition by toll-like receptor 5 (TLR5), and results in secretion of cytokines such as interleukin-8 (IL-8) into circulation (39). The secretion of IL-8 then stimulates an influx of neutrophils into the intestine in order to kill the *Salmonella*. Other cytokines that are produced, such as tumor necrosis factor-α (TNF-α), also are involved in the initiation of fever in response to pathogenic organisms. A study utilizing an *in vitro* culture of porcine intestinal cells found a reduction in the adherence of *Salmonella* in the presence of live yeast, and expression of pro-inflammatory genes in intestinal cell cultures in the presence of *Salmonella* was reduced, as well as secretion of interleukin-6 (IL-6), another cytokine involved in the development of fever (40). This suggests a reduction in binding of *Salmonella* to intestinal cells and reduced inflammation in the intestine in the presence of yeast. A reduction in inflammation in the gut may result in reduced systemic inflammation, thus the reduced body temperature observed in the YCW250 pigs. Sweeney, Collins (41) demonstrated that supplementing weaned pigs with β-glucans isolated from *Saccharomyces cerevisiae* reduced the population of *Enterobacteriaceae* within the intestine, specifically the ileum and the colon. Additionally, the authors found pro-inflammatory gene expression was reduced in the colon. Thus, changes in the microbial populations in the gut in response to the YCW supplement may have altered or reduced inflammatory responses. However, no differences in the *Salmonella* content within the specific gut tissues and digesta were found in the current study.

There are very few studies that have measured body temperature in yeast-supplemented pigs. A study utilizing an *E. coli* challenge in pigs supplemented with an autolyzed yeast found no differences in body temperature between the supplemented and control pigs (42). Additionally, pigs fed a yeast fermentation product and challenged with *Salmonella Typhimurium* found no differences in rectal temperature measured in supplemented vs. control pigs (25). Interesting, both the aforementioned challenges utilized greater bacterial challenge doses than the current study.

Cortisol is often activated concurrently with the immune system where it helps regulate and coordinate the inflammatory and anti-inflammatory responses of the immune system. Changes in cortisol have been previously observed in cattle supplemented with yeast products and exposed to an endotoxin challenge (8, 10), and changes in cortisol may provide insight into any changes observed in immune parameters. Limited differences in cortisol were observed during the study and cortisol concentrations remained very low throughout the study. In support of the current study, no difference was found in cortisol concentrations measured at various timepoints within the first 48 h post-*Salmonella* infection in finishing barrows (43). Thus, cortisol may not be an important variable to study in response to a live pathogen challenge.

Energy storage and utilization are very important factors associated with the proper functioning of the immune system, as the activated immune system requires a substantial amount of energy (44, 45). Additionally, changes in nutrient availability can also directly influence immune cell activation, thus affecting their fate and how the immune cells respond to pathogens (46). Thus, changes in glucose and NEFA concentrations may provide information on changes in energy utilization not only in response to YCW supplementation, but also in response to the *Salmonella* challenge itself. While glucose and NEFA concentrations differed between YCW treatments at a few time points post-challenge in the current study, there was no consistent effects of treatment on glucose or NEFA concentrations.

The biggest differences observed in the current study were related to hematology parameters. However, when analyzed closely, similar to the temperature response, the differences observed appear to be more related to the stress associated with the challenge administration rather than directly from the *Salmonella* infection. Specifically, an increase in total white blood cells, neutrophils, and the neutrophil:lymphocyte ratio occur between 6 and 18 h following the challenge. This is in the same time frame as the increase observed in intraperitoneal temperature. As discussed earlier, handling stress can increase intraperitoneal temperature, but can also increase concentrations of immune cells (47). Thus, it is likely that the increases observed in these particular immune cell parameters are not a result of a *Salmonella* infection, but rather due to the administration of the challenge. A study by Dubreuil, Farmer (47) reported similar changes in leukocyte profiles, including total leukocytes, neutrophils, lymphocytes and the N:L in pigs exposed to restraint stress for 5 min, in support of the data observed during the current study. Later during the challenge (30 to 36 h post-challenge), an increase in the neutrophil:lymphocyte ratio is observed in the YCW250 treatment group compared to Control. As reported earlier, an increase in neutrophil influx to the intestine is one of the first innate immune actions observed in response to *Salmonella* infection (39). Further, components of the yeast cell wall, such as β-glucans and mannan oligosaccharides, have been demonstrated to increase production of immune cell populations, including neutrophils and monocytes (5). While there is no statistical increase in neutrophil concentrations in the YCW250 group, lymphocyte concentrations were on average reduced in this treatment group compared to Control pigs. This may account for the treatment differences observed in the neutrophil:lymphocyte ratio during this time period. There were also small differences in monocyte and basophil concentrations between treatments; however, they are not likely of biological significance. Overall, it appears that hematological parameters were influenced more by the stress associated with administration of the challenge, and less influenced by the challenge itself. This is supported by the lack of treatment differences in fecal and tissue *Salmonella* counts observed in the current study.

## Conclusions

Weaned pigs supplemented with yeast cell wall had a reduction in the change in intraperitoneal temperature and decreased concentrations of various leukocyte populations in the post-challenge period following an oral *Salmonella Typhimurium* challenge. However, these responses appear to be more associated with a stress response from administration of the challenge, and less from the actual *Salmonella* infection. Additionally, there were limited effects on other parameters, including cortisol, glucose, NEFA, and *Salmonella* counts in feces and lower gastrointestinal tissues. Some of the observed changes in immune cell populations were similar to what has been observed previously in pigs supplemented with yeast products. However, overall there appear to be minimal effects of supplementation with the yeast cell wall product on the acute immune cell response to *Salmonella* challenge in weaned pigs. Additionally, the data suggest that different dosing rates of yeast cell wall may influence various immune cell populations.

## Ethics Statement

This study was carried out in accordance with the recommendations of the Guide for the Care and Use of Agricultural Animals in Research and Teaching and approved by the Institutional Animal Care and Use Committee at the Livestock Issues Research Unit.

## Author Contributions

NB, JAC, and JRC initiated the initial study design. NB, PB, JAC, and TC performed the study and collected data. Data was analyzed and the manuscript was written by NB. All authors reviewed drafts of the manuscript and provided approval prior to submission.

### Conflict of Interest Statement

JRC was employed by Phileo Lesaffre Animal Care. The remaining authors declare that the research was conducted in the absence of any commercial or financial relationships that could be construed as a potential conflict of interest.

## Abbreviations

ADG: Average daily gain
BW: Body weight
IL-1β: Interleukin-1β
IL-6: Interleukin-6
NEFA: non-esterified fatty acids
RBC: red blood cells
TNF-α: Tumor necrosis factor-α
TLR5: Toll-like receptor 5
YCW: Yeast cell wall
WBC: White blood cells.
