# Supplementary material for: Changes in the Hematological Variables in Pigs Supplemented With Yeast Cell Wall in Response to a Salmonella Challenge in Weaned Pigs
Source: Front Vet Sci. 2019 Jul 24;6:246. doi: 10.3389/fvets.2019.00246 (PMC6667829; doi:10.3389/fvets.2019.00246)
Supplement: Supplementary file 1 [file Data_Sheet_1.docx]

**Changes in the hematological variables in pigs supplemented with yeast cell wall in response to a *Salmonella* challenge in weaned pigs**

**Supplementary Table 1.** Summary of time effects serum cortisol, glucose and non-esterified fatty acid (NEFA) concentrations measured in pigs fed a control (Control) diet or supplemented with a yeast cell wall at 250 mg/kg (YCW250) or 500 mg/kg (YCW500), prior to administration of an oral *Salmonella Typhimurium* challenge on d 19.

|  |  | Time | |  |  |  |
| --- | --- | --- | --- | --- | --- | --- |
| Variable | TRT^1^ | -6 h | 0 h | SEM | Time | Interaction^2^ |
| Cortisol (ng/mL) | Control | 4.70 | 9.88 | 1.12 | **0.004** | 0.34 |
|  | YCW250 | 3.71 | 5.76 | 1.01 |  |  |
|  | YCW500 | 5.60 | 7.43 | 1.01 |  |  |
| Glucose (mg/dL) | Control | 91.04 | 89.79 | 4.25 | 0.23 | 0.53 |
|  | YCW250 | 98.65 | 90.66 | 4.25 |  |  |
|  | YCW500 | 88.67 | 87.58 | 4.07 |  |  |
| NEFA (mmol/L) | Control | 0.14 | 0.15 | 0.02 | 0.14 | 0.91 |
|  | YCW250 | 0.13 | 0.15 | 0.02 |  |  |
|  | YCW500 | 0.11 | 0.13 | 0.02 |  |  |
|  |  |  |  |  |  |  |

^1^TRT: Treatment

^2^Interaction: Treatment x time

**Supplementary Table 2.** Summary of time effects for complete blood cell count parameters measured in pigs fed a control (Control) diet or supplemented with a yeast cell wall at 250 mg/kg (YCW250) or 500 mg/kg (YCW500), prior to administration of an oral *Salmonella Typhimurium* challenge on d 19.

|  |  | Time | |  |  |  |
| --- | --- | --- | --- | --- | --- | --- |
| Variable | TRT^1^ | -6 h | 0 h | SEM | Time | Interaction^2^ |
| Red blood cells (10^6^/µL) | Control | 5.36 | 5.20 | 0.12 | 0.22 | 0.87 |
|  | YCW250 | 5.52 | 5.47 | 0.12 |  |  |
|  | YCW500 | 5.67 | 5.50 | 0.13 |  |  |
| Hemoglobin (g/dL) | Control | 8.82 | 8.56 | 0.18 | 0.26 | 0.84 |
|  | YCW250 | 8.87 | 8.82 | 0.18 |  |  |
|  | YCW500 | 8.92 | 8.73 | 0.17 |  |  |
| Hematocrit (%) | Control | 28.62 | 27.78 | 0.67 | 0.23 | 0.83 |
|  | YCW250 | 28.68 | 28.50 | 0.64 |  |  |
|  | YCW500 | 28.96 | 28.07 | 0.64 |  |  |
| Platelets (10^3^/µL) | Control | 321.55 | 328.17 | 27.29 | 0.56 | 0.83 |
|  | YCW250 | 377.92 | 408.50 | 26.13 |  |  |
|  | YCW500 | 400.75 | 401.58 | 26.13 |  |  |
| White blood cells (10^3^/µL) | Control | 18.87 | 19.25 | 1.37 | 0.95 | 0.90 |
|  | YCW250 | 16.73 | 16.88 | 1.32 |  |  |
|  | YCW500 | 17.52 | 16.79 | 1.32 |  |  |
| Neutrophils (10^3^/µL) | Control | 6.57 | 7.08 | 1.22 | 0.83 | 0.94 |
|  | YCW250 | 6.49 | 6.88 | 1.17 |  |  |
|  | YCW500 | 5.78 | 5.51 | 1.17 |  |  |
| Lymphocytes (10^3^/µL) | Control | 10.42 | 10.39 | 0.60 | 0.67 | 0.96 |
|  | YCW250 | 8.40 | 8.20 | 0.57 |  |  |
|  | YCW500 | 10.02 | 9.64 | 0.57 |  |  |
| N:L^3^ | Control | 0.65 | 0.70 | 0.16 | 0.78 | 0.91 |
|  | YCW250 | 0.77 | 0.87 | 0.16 |  |  |
|  | YCW500 | 0.64 | 0.61 | 0.16 |  |  |
| Monocytes (10^3^/µL) | Control | 1.56 | 1.57 | 0.12 | 0.76 | 0.97 |
|  | YCW250 | 1.54 | 1.50 | 0.11 |  |  |
|  | YCW500 | 1.42 | 1.37 | 0.11 |  |  |
| Eosinophils (10^3^/µL) | Control | 0.28 | 0.21 | 0.04 | 0.15 | 0.60 |
|  | YCW250 | 0.29 | 0.25 | 0.04 |  |  |
|  | YCW500 | 0.30 | 0.26 | 0.04 |  |  |
| Basophils (10^3^/µL) | Control | 0.04 | 0.01 | 0.02 | 0.96 | 0.17 |
|  | YCW250 | 0.01 | 0.05 | 0.02 |  |  |
|  | YCW500 | 0.01 | 0.00 | 0.02 |  |  |
|  |  |  |  |  |  |  |

^ab^Treatments with different superscripts within columns differ (*P* ≤ 0.05)

^1^TRT: Treatment

^2^Interaction: Treatment x time

^3^N:L: Neutrophil:lymphocyte ratio

**Supplementary Table 3.** Summary of time effects for serum cortisol glucose, and non-esterified fatty acid (NEFA) concentrations measured in pigs fed a control (Control) diet or supplemented with a yeast cell wall at 250 mg/kg (YCW250) or 500 mg/kg (YCW500), following administration of an oral *Salmonella Typhimurium* challenge on d 19.

|  |  | Time (h) | | | | | | | | | | | | | |  | |  |  |
| --- | --- | --- | --- | --- | --- | --- | --- | --- | --- | --- | --- | --- | --- | --- | --- | --- | --- | --- | --- |
| Variable | TRT^1^ | 0 | 6 | 12 | 18 | 24 | 30 | 36 | 42 | 48 | 54 | 60 | 66 | 72 | SEM | | Time | | TRT^*^Time |
| Cortisol (ng/mL) | Control | 9.88^a^ | 4.76 | 2.73 | 5.53 | 1.59^b^ | 1.97^b^ | 4.48 | 6.47 | 4.75 | 2.00 | 1.87 | 1.71 | 3.73 | 1.10 | | **<0.01** | | **0.03** |
|  | YCW250 | 5.76^b^ | 3.63 | 4.23 | 4.65 | 2.98 | 4.00 | 4.83 | 4.56 | 6.61 | 3.45 | 2.05 | 3.28 | 3.35 | 1.02 | |  | |  |
|  | YCW500 | 7.43^b^ | 5.50 | 4.59 | 3.30 | 4.15^a^ | 4.65^a^ | 5.37 | 7.16 | 4.27 | 3.37 | 3.85 | 3.89 | 3.27 | 0.91 | |  | |  |
| Glucose (mg/dL) | Control | 89.79 | 79.45^b^ | 90.91 | 84.61 | 82.99 | 84.49 | 97.66 | 97.93 | 107.32 | 101.27 | 107.65 | 98.44 | 96.57 | 4.46 | | **<0.01** | | **0.01** |
|  | YCW250 | 90.66 | 93.19^a^ | 101.40 | 93.41^a^ | 85.09 | 79.66 | 90.60 | 91.79 | 98.45 | 96.16 | 93.69 | 100.40 | 97.05 | 5.33 | |  | |  |
|  | YCW500 | 87.58 | 85.58 | 97.81 | 81.16^b^ | 81.48 | 91.87 | 101.62 | 104.78 | 103.52 | 92.45 | 102.73 | 100.62 | 81.93 | 4.46 | |  | |  |
| NEFA (mmol/L) | Control | 0.15 | 0.25 | 0.16 | 0.21 | 0.22 | 0.13 | 0.16 | 0.13 | 0.15 | 0.13 | 0.12 | 0.13 | 0.23 | 0.02 | | 0.06 | | 0.07 |
|  | YCW250 | 0.15 | 0.13 | 0.13 | 0.15 | 0.17 | 0.12 | 0.13 | 0.13 | 0.15 | 0.15 | 0.11 | 0.12 | 0.12 | 0.03 | |  | |  |
|  | YCW500 | 0.13 | 0.16 | 0.16 | 0.13 | 0.13 | 0.14 | 0.13 | 0.16 | 0.18 | 0.16 | 0.15 | 0.12 | 0.14 | 0.02 | |  | |  |
|  |  |  |  |  |  |  |  |  |  |  |  |  |  |  |  | |  | |  |

^ab^Treatments with different superscripts within columns differ (*P* ≤ 0.05)

^1^TRT: Treatment

^2^Interaction: Treatment x time

**Supplementary Table 4.** Summary of time effects for complete blood cell count parameters measured in pigs fed a control (Control) diet or supplemented with a yeast cell wall at 250 mg/kg (YCW250) or 500 mg/kg (YCW500), following administration of an oral *Salmonella Typhimurium* challenge on d 19.

|  |  | Time (h) | | | | | | | | | | | | | | | | | | | | | | | | | |  | | |  |  |  |  |  |
| --- | --- | --- | --- | --- | --- | --- | --- | --- | --- | --- | --- | --- | --- | --- | --- | --- | --- | --- | --- | --- | --- | --- | --- | --- | --- | --- | --- | --- | --- | --- | --- | --- | --- | --- | --- |
| Variable | TRT^1^ | | 0 | | 6 | | 12 | | 18 | | 24 | | 30 | | 36 | | 42 | | 48 | | 54 | | 60 | | 66 | | 72 | | | SEM | | | Time | TRT*  Time |  |
| RBC^3^ (10^6^/µL) | Control | | 5.20 | 5.17 | | 5.11 | | 5.29 | | 5.15 | | 5.23 | | 5.15 | | 5.07 | | 5.02 | | 5.13 | | 5.16 | | 5.02 | | 4.99 | | | 0.13 | | | **<0.01** | | 0.81 | |
|  | YCW250 | | 5.47 | 5.56 | | 5.49 | | 5.33 | | 5.19 | | 5.45 | | 5.32 | | 5.29 | | 5.04 | | 5.34 | | 5.22 | | 5.04 | | 5.16 | | | 0.14 | | |  | |  | |
|  | YCW500 | | 5.50 | 5.38 | | 5.37 | | 5.59 | | 5.28 | | 5.17 | | 5.08 | | 5.23 | | 4.96 | | 5.06 | | 5.19 | | 4.97 | | 4.96 | | | 0.13 | | |  | |  | |
| Hemoglobin (g/dL) | Control | | 8.56 | 8.35 | | 8.41 | | 8.64 | | 8.43 | | 8.52 | | 8.39 | | 8.28 | | 8.31 | | 8.28 | | 8.33 | | 8.18 | | 8.08 | | | 0.20 | | | **<0.01** | | 0.93 | |
|  | YCW250 | | 8.82 | 8.70 | | 8.75 | | 8.48 | | 8.28 | | 8.51 | | 8.41 | | 8.33 | | 8.08 | | 8.43 | | 8.29 | | 8.03 | | 8.22 | | | 0.23 | | |  | |  | |
|  | YCW500 | | 8.73 | 8.40 | | 8.47 | | 8.68 | | 8.22 | | 8.05 | | 8.00 | | 8.06 | | 7.78 | | 7.95 | | 8.04 | | 7.77 | | 7.87 | | | 0.19 | | |  | |  | |
| Hematocrit (%) | Control | | 27.78 | 27.26 | | 27.35 | | 28.81 | | 28.00 | | 27.84 | | 27.75 | | 27.45 | | 27.28 | | 27.33 | | 28.00 | | 26.93 | | 27.04 | | | 0.73 | | | **<0.00** | | 0.87 | |
|  | YCW250 | | 28.50 | 28.18 | | 28.50 | | 27.58 | | 26.92 | | 27.19 | | 27.12 | | 27.58 | | 25.98 | | 27.07 | | 26.79 | | 26.03 | | 26.93 | | | 0.81 | | |  | |  | |
|  | YCW500 | | 28.07 | 26.89 | | 27.28 | | 28.59 | | 26.95 | | 25.78 | | 26.00 | | 26.68 | | 25.30 | | 25.53 | | 26.53 | | 25.77 | | 25.65 | | | 0.69 | | |  | |  | |
| Platelets (10^3^/µL) | Control | | 328.17 | 329.75 | | 341.42 | | 339.42 | | 344.17 | | 359.92 | | 354.82 | | 346.91 | | 369.17 | | 372.45 | | 391.00 | | 399.00 | | 397.50 | | | 26.13 | | | 0.09 | | 0.99 | |
|  | YCW250 | | 408.50 | 391.92 | | 375.08 | | 364.50 | | 352.08 | | 368.60 | | 399.22 | | 390.50 | | 383.64 | | 420.91 | | 398.91 | | 423.36 | | 429.91 | | | 28.01 | | |  | |  | |
|  | YCW500 | | 401.58 | 386.92 | | 400.58 | | 388.83 | | 385.58 | | 400.25 | | 387.50 | | 380.83 | | 371.00 | | 404.00 | | 411.25 | | 412.55 | | 405.55 | | | 23.88 | | |  | |  | |
| WBC^4^ (10^3^/µL) | Control | | 19.25 | 29.15^a^ | | 28.04^a^ | | 24.40^a^ | | 19.79 | | 19.06 | | 18.67 | | 18.70 | | 19.94 | | 19.82 | | 20.36 | | 19.05 | | 16.86 | | | 1.84 | | | **<0.01** | | **0.03** | |
|  | YCW250 | | 16.88 | 18.12^b^ | | 17.77^c^ | | 15.68^b^ | | 17.82 | | 18.46 | | 18.80 | | 19.98 | | 16.81 | | 18.25 | | 17.44 | | 17.71 | | 16.99 | | | 1.94 | | |  | |  | |
|  | YCW500 | | 16.79 | 22.02^b^ | | 22.72^b^ | | 21.54^a^ | | 18.16 | | 17.09 | | 16.52 | | 17.57 | | 17.22 | | 17.76 | | 16.75 | | 17.30 | | 18.23 | | | 1.68 | | |  | |  | |
| Neutrophils (10^3^/µL) | Control | | 7.08 | 18.75^a^ | | 15.24^a^ | | 11.19^a^ | | 7.16 | | 7.27 | | 6.84 | | 6.99 | | 7.70 | | 8.96 | | 7.67 | | 6.96 | | 6.56 | | | 1.66 | | | **<0.01** | | **0.02** | |
|  | YCW250 | | 6.88 | 9.18^b^ | | 8.20^b^ | | 6.45^b^ | | 8.67 | | 9.67 | | 9.27 | | 9.86 | | 7.50 | | 7.95 | | 7.13 | | 6.53 | | 6.22 | | | 1.75 | | |  | |  | |
|  | YCW500 | | 5.51 | 11.80^b^ | | 11.05^b^ | | 9.34 | | 7.07 | | 7.36 | | 7.14 | | 7.12 | | 6.49 | | 7.67 | | 6.24 | | 6.88 | | 7.71 | | | 1.52 | | |  | |  | |
| Lymphocytes (10^3^/µL) | Control | | 10.39 | 8.67 | | 9.62 | | 10.78 | | 10.50 | | 9.82 | | 9.80 | | 9.60 | | 10.05 | | 8.89 | | 10.36 | | 9.78 | | 8.44 | | | 0.68 | | | 0.25 | | 0.52 | |
|  | YCW250 | | 8.20 | 7.45 | | 7.82 | | 7.55 | | 7.35 | | 7.27 | | 7.47 | | 7.79 | | 7.48 | | 8.36 | | 8.31 | | 8.81 | | 8.53 | | | 0.71 | | |  | |  | |
|  | YCW500 | | 9.64 | 8.79 | | 9.58 | | 10.17 | | 9.22 | | 8.26 | | 7.81 | | 8.68 | | 8.86 | | 8.47 | | 8.79 | | 8.57 | | 8.60 | | | 0.62 | | |  | |  | |
| N:L^5^ | Control | | 0.70 | 2.41^a^ | | 1.84^a^ | | 1.18 | | 0.71 | | 0.77^b^ | | 0.72^b^ | | 0.76 | | 0.80 | | 1.04 | | 0.77 | | 0.72 | | 0.80 | | | 0.26 | | | **<0.01** | | **0.03** | |
|  | YCW250 | | 0.87 | 1.20^b^ | | 1.06^b^ | | 0.86 | | 1.24 | | 1.51^a^ | | 1.53^a^ | | 1.22 | | 1.00 | | 0.97 | | 0.88 | | 0.77 | | 0.76 | | | 0.27 | | |  | |  | |
|  | YCW500 | | 0.61 | 1.57^b^ | | 1.41 | | 1.02 | | 0.82 | | 0.99 | | 1.10 | | 0.91 | | 0.77 | | 0.97 | | 0.73 | | 0.92 | | 0.89 | | | 0.24 | | |  | |  | |
| Monocytes (10^3^/µL) | Control | | 1.57 | 1.61 | | 1.82 | | 2.02 | | 1.81 | | 1.67 | | 1.73 | | 1.80 | | 1.90 | | 1.70 | | 2.04 | | 2.02 | | 1.65 | | | 0.15 | | | **<0.01** | | 0.16 | |
|  | YCW250 | | 1.50 | 1.19 | | 1.37 | | 1.36 | | 1.52 | | 1.21 | | 1.65 | | 1.90 | | 1.61 | | 1.64 | | 1.64 | | 2.00 | | 1.94 | | | 0.16 | | |  | |  | |
|  | YCW500 | | 1.37 | 1.28 | | 1.50 | | 1.70 | | 1.56 | | 1.22 | | 1.29 | | 1.47 | | 1.53 | | 1.30 | | 1.42 | | 1.54 | | 1.60 | | | 0.14 | | |  | |  | |
| Eosinophils (10^3^/µL) | Control | | 0.21 | 0.12 | | 1.35 | | 0.40 | | 0.31 | | 0.30 | | 0.28 | | 0.29 | | 0.24 | | 0.25 | | 0.27 | | 0.24 | | 0.20 | | | 0.21 | | | 0.08 | | 0.87 | |
|  | YCW250 | | 0.25 | 0.25 | | 0.31 | | 0.27 | | 0.22 | | 0.24 | | 0.23 | | 0.27 | | 0.21 | | 0.29 | | 0.31 | | 0.31 | | 0.27 | | | 0.23 | | |  | |  | |
|  | YCW500 | | 0.26 | 0.14 | | 0.58 | | 0.32 | | 0.29 | | 0.24 | | 0.21 | | 0.29 | | 0.30 | | 0.23 | | 0.28 | | 0.29 | | 0.27 | | | 0.20 | | |  | |  | |
| Basophils (10^3^/µL) | Control | | 0.01 | 0.00 | | 0.01 | | 0.01 | | 0.01 | | 0.01 | | 0.01 | | 0.01 | | 0.04 | | 0.02 | | 0.02 | | 0.06 | | 0.01 | | | 0.04 | | | 0.33 | | 0.31 | |
|  | YCW250 | | 0.05 | 0.05 | | 0.07 | | 0.06 | | 0.07 | | 0.07 | | 0.19 | | 0.16 | | 0.01 | | 0.01 | | 0.05 | | 0.07 | | 0.03 | | | 0.04 | | |  | |  | |
|  | YCW500 | | 0.00 | 0.01 | | 0.01 | | 0.02 | | 0.01 | | 0.01 | | 0.09 | | 0.01 | | 0.04 | | 0.07 | | 0.01 | | 0.01 | | 0.04 | | | 0.03 | | |  | |  | |
|  |  | |  | |  | |  | |  | |  | |  | |  | |  | |  | |  | |  | |  | |  | | |  | | |  |  |  |

^ab^Treatments with different superscripts within columns differ (*P* ≤ 0.05)

^1^TRT: Treatment

^2^Interaction: Treatment x time

^3^RBC: Red blood cells

^4^WBC: White blood cells

^5^N:L: Neutrophil:lymphocyte ratio
